# Robotics improves alignment accuracy and reduces early revision rates for UKA in the hands of low-volume UKA surgeons

**DOI:** 10.1007/s00402-021-04114-5

**Published:** 2021-08-18

**Authors:** Peter Savov, Lars-Rene Tuecking, Henning Windhagen, Tilman Calliess, Max Ettinger

**Affiliations:** 1 Department of Orthopedic Surgery, Hannover Medical School, Anna-von-Borries-Strasse 1-7, 30625 Hanover, Germany; 2 Articon CHRISTENORTO AG, Schänzlistrasse 39, 3013 Bern, Switzerland

**Keywords:** UKA, Robotic-assisted surgery, Imageless, Image-based, Low volume

## Abstract

**Purpose:**

It is known that in uni-compartmental knee arthroplasty (UKA) low-volume surgeons have a higher complication and revision rate than high-volume surgeons. Further, robotic-assisted UKA leads to lower early revision rate as well as fewer limb and joint line outliers compared to conventional UKA. The purpose of this study was to retrospectively analyze the outliers’ and revision rate of low-volume UKA surgeons with different robotic systems at short-term follow-up.

**Methods:**

In this case–control study, 103 robotic-assisted UKAs were included. The procedures were performed between 2016 and 2019 from two low-volume UKA surgeons with an imageless (IL) (63 patients) and image-based (IB) (40 patients) robotic system. Alignment outliers, joint line (JL) reconstruction, complication and revision rates of the two different robotic systems were analyzed. The minimum follow-up was two years. Outliers were defined as a postoperative valgus malalignment greater than 182°. The surgery time for all procedures was evaluated.

**Results:**

The overall revision rate was 3.9% (4 of 103). Two occurred in the IB group (5.0%) and two in the IL group (3.2%). No valgus malalignment outliers were observed in both groups. The mean JL was not distalized by more than 2 mm in both groups (IL: 1.3 ± 1.6 mm vs. IB: 1.8 ± 0.9 mm, *p* value 0.08). The IL procedures had a significant lower mean surgery time (55 ± 13 min vs. 68 ± 14, *p* value 0.001).

**Conclusion:**

Robotic-assisted UKA is a safe procedure in the hand of low-volume UKA surgeons. Robotic-assisted UKA minimizes overcorrection into valgus mal-alignment. Low revision rates are observed at short-term follow-up for robotic-assisted UKA. The choice of the different robotic systems has no impact on the outcome.

## Introduction

Uni-compartmental knee arthroplasty (UKA) is an established method for the treatment of isolated anteromedial osteoarthritis (OA) [1]. The indications for this procedure are clearly defined [2, 3]. If positioned correctly, UKA may lead to superior functional outcomes to total knee arthroplasty (TKA) [4, 5]. The major benefit of medial UKA over TKA is to closely restore normal knee kinematics. It is known that an alteration of parameters, e.g., joint line height, varus alignment or tibial slope, has a negative impact on the outcome and kinematics [6]. It is evident that a slight varus under-correction leads to superior postoperative results [7]. An improved accuracy is required for an optimal positioning of the implant.

Image-based (IB) robotic arm-assisted UKA and imageless (IL) robotic hand-piece-assisted procedures are well established and known to deliver high precision [8–12]. Both systems help the surgeon implementing the preoperative plan with a real-time monitoring during the surgery. Various studies have shown advantages in implant alignment and soft tissue balancing compared to conventional surgery [8, 10, 11]. With IB robotic arm- or IL robotic hand-piece-assisted UKA, less distalization of the joint line is desired [8]. However, differences in alignment and joint line reconstruction between IB and IL robotic systems have not yet been investigated.

It is known, that the survival rate of UKA is inferior to TKA [13–15]. Wrong indication, malalignment of the limb as well as young patient age are risk factors for an early reoperation [13, 16]. However, with the introduction of robot-assisted surgery, a decrease in revision rates has already been shown, particularly in the Australian registry [17, 18]. Improved implant positioning and a reduction of outliers are considered as relevant factors. Further, the hospital case number has a significant influence on the 5-year survival. Low-volume hospitals with less than 25 UKAs per year have twice the risk for a surgical revision in contrast to high-volume hospitals with more than 100 cases per year [19]. However, there are currently no data on the impact of robotic-assisted surgery on the revision rates of low-volume UKA surgeons.

The aim of this study was (1) to demonstrate that both image-based robotic arm-assisted and imageless robotic hand-piece-assisted UKA are able to improve surgical accuracy in UKA (2) to examine whether robotics reduces the risk for overcorrection into valgus alignment (3) to evaluate the robotic-assisted UKA revision rate at short-term follow-up of low-volume UKA surgeons. The primary hypothesis was that robotic-assisted UKA leads to an accurate joint line reconstruction in the hands of low-volume UKA surgeons.

## Materials and methods

### Patients

In this retrospective study, 103 robotic-assisted UKA cases were included. In 40 cases, IB robotic arm-assisted surgery was performed (Restoris MCK, MAKO^®^, Stryker Corporation, Kalamazoo, MI). The other 63 patients received an IL robotic hand-piece-assisted UKA (NAVIO^®^ Journey 1 UKA, Smith and Nephew, Memphis, USA). The IB robotic arm-assisted system was introduced to the authors’ hospital in September 2016, the IL robotic hand-piece system in March 2018. The surgeries were carried out between September 2016 and January 2019. All robotic-assisted UKAs performed in this period were included in this study. Patients with an allergy against nickel or cobalt-chrome were chosen for the IL robotic system using an implant with oxinium coating. Furthermore, patients refusing a preoperative planning CT scan or were not able to undergo the CT scan were also chosen for the IL robotic system. All patients had a medial OA. The procedures were performed by two single surgeons who performed less than 5 UKAs per year before 2016. However, the surgeons performed more than 50 total knee arthroplasties in the 12 months immediately prior to the first study case. The minimum follow-up time was two years. Revisions were analyzed. Inclusion criteria were isolated bone-on-bone arthritis of the medial compartment, full thickness of lateral cartilage, physiological medial collateral and anterior cruciate ligaments [24] and a BMI below 35.

For the robotic arm-assisted group, a pre-operative CT scan was used to construct a 3D bone model of each patient. The position of the prosthesis was planned concerning the extension and flexion gaps through the whole range of motion with respect to the bony anatomy. In the imageless robotic hand-piece group, the 3D bone model of the knee joint was mapped and reconstructed during the operation. Afterwards, the position of the prosthesis was planned like the UKAs in the IB group.

### Measurements

**Fig. 1:**
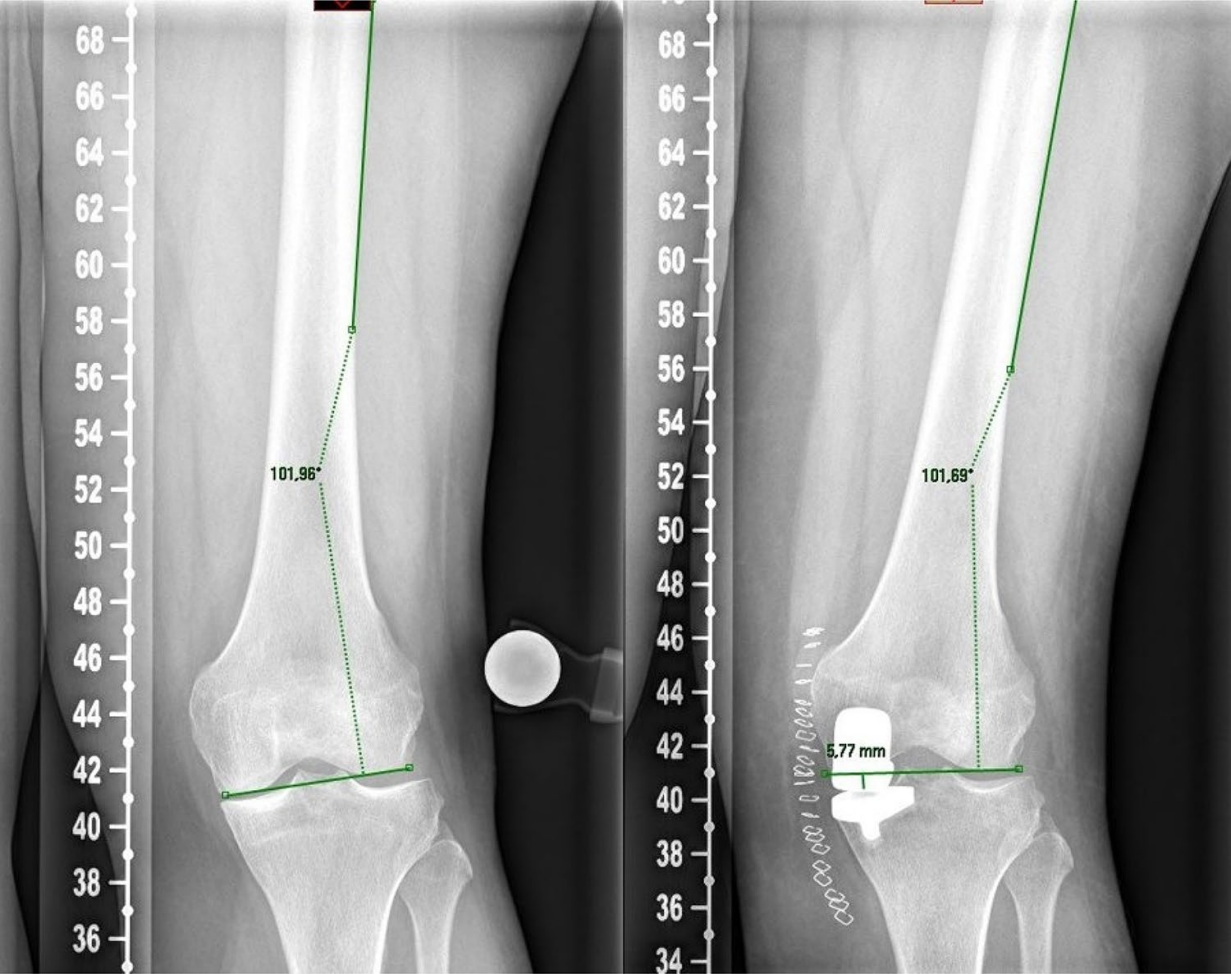
Measurement of the joint line height with the method described by Henry et al.

Standard radiographs were performed preoperatively and three months postoperatively. Radiographs included a weight-bearing long leg view and a lateral view. The joint line height (JL), the hip knee ankle (HKA), medial proximal tibial angle (MPTA) and the tibial slope (Slope) were measured. The joint line was measured as the angle between the lateral femoral cortex and a line through the most distal point of the femoral condyles. On postoperative radiographs, the same angle is applied with the reference to the lateral femoral cortex. The line through the femoral condyles is referenced to the non-operated lateral condyle. The distance from the most distal point of the implant to this line was measured. These results were defined as the joint line alteration (Fig. 1). This method was described by Herry et al. [8]. Two investigators (LT and PS) measured these parameters twice independently from each other. The intra- and inter-observer accuracy was measured. The mean JL distalization was compared between the two groups. Postoperative outliers of limb alignment and joint line alteration were compared. Limb outliers were defined as a postoperative valgus mal-alignment greater than 182°. JL outliers were defined as a postoperative distalization of more than 2 mm. The surgery time from skin incision to suture was evaluated from all patients.

### Statistics

The software Carestream PACS (Carestream Health Deutschland GmbH, Stuttgart, Germany) was used for all measurements. The measurement accuracy was at one decimal. The statistical evaluation was carried out with GraphPad Prism Version 7 (GraphPad Inc., San Diego, CA, USA). The Student’s *t* test was used to calculate significant differences between the two groups. A power analysis was conducted on the base of the joint line alteration; based on previous literature, an alteration of the joint line of 1.5 mm (SD 2.5 mm) was expected in both robotic groups [8, 20]. A difference in joint line alteration of 2 mm (SD 2.5 mm) was defined as clinical significant [8]. The GPower software (HHU Düsseldorf, Düsseldorf, Germany) was used to determine the sample size. The calculated sample size of each group was at least 35 patients to achieve a power of 95% (*a* = 0.05).

## Results

The demographics and the mean surgery time of both groups are listed in Table 1. The HKA and MPTA change significantly in both groups postoperatively. The joint line was slightly distalized in both groups, there were no significant differences (Table 2). Within the time of follow-up, two revisions occurred in the IB group (5.0%) and two in the IL group (3.2%). The overall revision rate was 3.9% (4 of 103). Two patients had an early aseptic loosening, one patient was revised to a TKA due to ACL instability after trauma and one due to a fracture of the tibia plateau after jumping on a trampoline. No outliers for valgus overcorrection with more than 2° of postoperative valgus were observed in both groups. No significant differences were observed in JL distalization of more than 2 mm between the two groups (32.5% IB vs. 33.3% IL, *p* = 0.553) (Table 3). The inter-observer accuracy was 0.922 and the intra-observer accuracy was 0.953.

**Table 1:** Demographics and surgery time of the image-based (IB) and image-less (IL) robotic group

*n* = 103 (IB: 40; IL: 63)	IB Robot	IL Robot	*P* value
Age	69 (± 11)	64 (± 8.9)	0.008
BMI	28 (± 5.3)	28 (± 3.3)	0.869
Follow up (Months)			
Mean	38	30	
Min	24	24	
Max	52	39	
Surgery time (min)			
Mean (SD)	68 (± 14)	55 (± 13)	0.001
Min	42	36	
Max	90	83

**Table 2:** The pre- and postoperative mean

	IB Robotic system			IL Robotic system		
	pre	post	*P* value	pre	post	*P* value
HKA (°)						
Mean	175	178	< 0.001*	175	178	< 0.002*
SD	± 2.7	± 2.3		± 3.5	± 2.4	
Min	170	171		167	170	
Max	180	182		180	182	
Slope (°)						
Mean	4.0	4.8	0.075	4.5	5.3	0.121
SD	± 2.2	± 1.5		± 3.0	± 2.5	
Min	0.4	1.9		0	0	
Max	8.4	7.0		11	11	
JL distalization (mm)						
Mean		1.8			1.3	0.080
SD		± 0.9			± 1.6	
Min		0			−2.4	
Max		3.7			5.7	
MPTA (°)						
Mean	87	89	0.001*	87	89	< 0.001*
SD	± 2.3	± 1.1		± 2.1	± 1.6	
Min	83	87		81	87	
Max	90	91		91	93	

Standard deviation (*SD*) and range of the hip knee ankle (*HKA*), medial proximal tibia angle (*MPTA*) and the posterior tibial slope (Slope) of the image-based (*IB*) robotic and image-less (*IL*) robotic group are listed. Differences between the pre- and postoperative means were analyzed. The postoperative joint line distalization of the IB and IL group is not significant different

**Table 3:** The overall revision rate and outliers of both robotic-assisted groups

		IB robotic group	IL robotic group	*P* value
Revision	5.0%		3.2%	0.637
	2/40		2/63	
Valgus malalignment	0%		0%	0.999
Significant JL distalization	32.5%		33.3%	0.553
	21/63		13/40	

## Discussion

The most important finding of this study is that IB and IL robotic-assisted UKA provide equal radiological results. Robotic-assisted UKA of low-volume surgeons leads to low revision rates at short-term follow-up. Furthermore, there were no outliers for valgus malalignment in both groups.

The alternation of the natural joint line has an effect on the mechanics and kinematics of the joint. Biomechanical studies showed a higher load on the tibial tray with a deep tibia resection [21–23]. Joint space elevation leads to higher strains in the contralateral femorotibial compartment, thus progression of OA in the lateral compartment might occur [6]. In contrast to that, a distalization of the joint line of 4 mm increases the tibial strain up to 13 and 35%. In addition, it is known that a too distal tibia placement correlates with less resistance to compression forces. Early loosening of the tibial component may be the consequence [24]. Another benefit of an anatomical reconstruction of the joint line is the potential conversion of an UKA into a TKA. Distalization of the joint line accompanies with more bone loss and a potential need for augmentation [8, 25]. In a retrospective multicenter study of 559 medial UKAs, Chatellard et al. determined that a severe distalization of the joint is associated with decreased prosthesis survival [6]. Herry et al. reported postoperative findings after UKA with robotic-assisted surgery with the NAVIO system and a conventional technique. The distalization of the joint line in the robotic group was 1.4 mm. The range goes from −3 to 6 mm [8]. Klasan et al. reported a distalization of the joint line with the MAKO system of 1.5 mm (range: −0.6 mm to 4 mm) [20]. These results reflect our findings. We measured a mean distalization of the joint line in the IB robotic arm group of about 1.8 mm (range: 0 to 3.7 mm) compared to the IL robotic hand-piece group of about 1.3 mm (range: −2.5 to 5.7) (Table 2). All reported results of robotic-assisted UKA are located within 2 mm distalization. Thus, the joint line was reconstructed precisely. The scattering of the values with the IB robotic system is lower compared to the IL robotic system both in literature and in this study. However, both groups present about 33% outlier of joint line distalization of more than 2 mm.

The revision rate of UKA is one of the main issues compared to TKA. The early as well as the late revision rate are significantly higher [14]. Main reasons are aseptic loosening and malalignment following implant mal-positioning [6, 26]. It is known that experienced UKA surgeons with a high-case volume per year have a significant lower revision rate and a better clinical outcome [27–30]. Baker et al. showed a revision rate of 2% for surgeons with more than 100 Oxford UKAs per year after 2 years. Surgeons with less than 25 UKAs per year had a revision rate of 5% [29]. Mohammad et al. presented similar results after 2 years. Surgeon with less than 10 Oxford UKAs per year had a lower survival rate than surgeons with more than 30 UKAs per year (96% vs. 99%) [30]. An analysis of the largest German health insurance provider of 20,946 UKAs showed that the revision rate after 5 years in hospitals with less than 25 UKAs per year was twice higher in clinics with more than 100 UKAs per year [19]. These data are additionally confirmed by the German arthroplasty registry [13]. The revision rate of hospitals with less than 30 UKAs per year is about 7% after two years. In contrast to that, centers with more than 100 UKAs per year have a revision rate of 3% after two years. However, data from hospitals cannot be fully compared with those from individual surgeons. Furthermore, most of the surgeons are unable to meet a high-case volume of UKAs per year [31]. In this context, robotic-assisted surgery could be an alternative. The precision of robotic-assisted UKA compared to the manual technique has meanwhile been proven. Due to this higher accuracy of implant positioning, revision rates of UKAs are decreasing. The most used UKA implant in Australia in the recent years was the IB robotic-assisted Restoris MCK^®^. The Australian registry showed the lowest revision rates for this implant after three years [17, 18]. The revision rate of this study is comparable to the data of the registries and literature. Mergenthaler et al. reported a revision rate with the IL robotic system of 4% after a minimum follow-up of one year [32]. Cool et al. were able to show a revision rate of even less than 1% after two years [33]. We demonstrate an overall revision rate of 3.9% (4 of 103) with robotic-assisted UKA after two years. There was no significant difference between the IB and IL robotic systems. Both surgeons performed an average of less than 30 UKAs per year in the period from 2016 to 2019. This study demonstrates that low-volume robotic-assisted UKA is a safe procedure.

It is known that a slight varus under-correction of the overall limb alignment and the MPTA leads to superior results in UKA [7]. An overcorrection to valgus alignment triggers an early osteoarthritis of the lateral compartment as well as chronical medial collateral ligament (MCL) pain [6, 34, 35]. Preoperative risk factors for postoperative valgus alignment are, e.g., a smaller LDFA and a higher MPTA [36]. These valgus-associated morphotypes are particularly difficult to treat with conventional mobile bearing UKAs. In this study, the mean postoperative MPTA was identical in between groups with 89°, whereas the standard deviation was slightly higher in the IL robotic group. This might indicate a higher accuracy in the IB robotic group, whereas the difference is within the range of measurement inaccuracy. In both groups, the MPTA was in the radiological safe zone [6]. The mean postoperative HKA in the IB robotic arm group was 178° varus and 178° varus in the IL robotic hand-piece group. The range in the IB group was 171° to 181° and in the IL group 170° to 182°. No overcorrection to a valgus alignment (HKA > 182°) was observed in both groups (Table 3). With robotic-assisted techniques, the surgeon has objective control over the postoperative limb alignment. These results are comparable to the robotic literature [37]. Batailler et al. reported a postoperative HKA of 175.2° (range: 170.8–185.6°) and a 16% limb outlier rate with the IL robotic system [9]. Outliers were defined as a HKA > 180° or < 176°. An explicit calculation of valgus overcorrection did not happen. The pre- and postoperative MPTA was not specified. Gaudiani et al. and Kayani et al. reported similar results with the IB robotic system. The mean postoperative HKA was 177.2° and 178.4°, respectively [10, 38]. The MPTA was not measured of both authors. The choice of robotic system does not seem to have any impact on the postoperative alignment.

The tibial slope has critical impact on knee kinematics and bone-quality [38]. Small et al. determined in a biomechanical analysis that a 3° of slope has the best balanced strain in response to loading after UKA [23]. The aim of robotic-assisted UKA is to restore the natural knee kinematics. In order to that, the reconstruction of the natural tibial slope is crucial. Our results are within these findings. The mean postoperative slopes of both groups did not change significantly (Table 2). The mean difference between the pre- and postoperative slope is within 1° in both groups. Our data are comparable to recent literature. In this respect, Gaudiani et al. reported a mean postoperative slope of 2.76° with the same IL robotic system [38]. Kayani et al. reported a mean postoperative slope of 1.94° with the IB and Batailler et al. reported a mean postoperative slope of 3.6° with the IL robotic system [9, 10].

The surgery time was significant different between both groups. The mean surgery time of the IL group was 55 min and on average 13 min faster than the IB group (68 min). Our results for the IB robotic system are slightly different compared to those to the literature. Kayani et al. reported a mean surgery time of 62 min after finishing the learning curve [10]. Leelasestaporn et al. reported a mean surgery time of 98 min for the IL system and 82.5 min for the IB system [39]. However, the total sample size of this cohort was 33 and the surgeries were performed over a period of 3 years. Further, no information was provided regarding the learning curve of the surgeon. In order to that, these results are not comparable to our data. In our opinion, the software steps and the burring procedure of the IL system take less time but the experience of the surgeon is significantly more crucial.

There are some limitations in this study. First, this study is a retrospective case–control study. In order to that, a prospective randomized trial could provide a higher level of evidence. An additional control group of high-volume conventional UKA surgeons would provide more interesting results. However, analysis of limb alignment and joint line reconstruction could be done retrospectively on early postoperative radiographs. The measurements were done on digital 2D radiographs. It is known that long leg radiographs only show good reliability compared to CT measurements [40]. Second, the measurements were based on bony landmarks. However, the aim is to reconstruct the cartilage. Third, this is a short-term study with a follow-up of two years. Furthermore, no clinical data is presented in this study and there is no conventional control group. Finally, the groups were not matched. Thus, confounders like BMI, gender and age could have an influence on the results.

## Conclusion

Robotic-assisted UKA is a safe procedure in the hand of low-volume UKA surgeons. Robotic-assisted UKA minimizes overcorrection into valgus mal-alignment. Low revision rates are observed at short-term follow-up for robotic-assisted UKA. The choice of the different robotic systems has no impact on the outcome.
